# Image reconstruction utilizing median filtering applied to elastography

**DOI:** 10.1186/s12938-019-0641-6

**Published:** 2019-03-12

**Authors:** Rubem P. Carbente, Joaquim M. Maia, Amauri A. Assef

**Affiliations:** 1 Electrical Engineering Department and the Graduate School of Electrical Engineering (DAELT), Federal University of Technology–Paraná (UTFPR), Curitiba, PR Brazil; 2 Electrical/Electronic Engineering Department and the Graduate School of Electrical Engineering and Applied Computer Sciences (DAELT–DAELN–CPGEI), Federal University of Technology–Paraná (UTFPR), Curitiba, PR Brazil

**Keywords:** Ultrasound, Shear wave, Elastography, Signal processing, Ultrafast imaging

## Abstract

**Background:**

The resources of ultrafast technology can be used to add another analysis to ultrasound imaging: assessment of tissue viscoelasticity. Ultrafast image formation can be utilized to find transitory shear waves propagating in soft tissue, which permits quantification of the mechanical properties of the tissue via elastography. This technique permits simple and noninvasive diagnosis and monitoring of disease.

**Methods:**

This article presents a method to estimate the viscoelastic properties and rigidity of structures using the ultrasound technique known as shear wave elasticity imaging (SWEI). The Verasonics Vantage 128 research platform and L11-4v transducer were used to acquire radio frequency signals from a model 049A elastography phantom (CIRS, USA), with subsequent processing and analysis in MATLAB.

**Results:**

The images and indexes obtained reflect the qualitative measurements of the different regions of inclusions in the phantom and the respective alterations in the viscoelastic properties of distinct areas. Comparison of the results obtained with this proposed technique and other commonly used techniques demonstrates the characteristics of median filtering in smoothing variations in velocity to form elastographic images. The results from the technique proposed in this study are within the margins of error indicated by the phantom manufacturer for each type of inclusion; for the phantom base and for type I, II, III, and IV inclusions, respectively, in kPa and percentage errors, these are 25 (24.0%), 8 (37.5%), 14 (28.6%), 45 (17.8%), and 80 (15.0%). The values obtained using the method proposed in this study and mean percentage errors were 29.18 (− 16.7%), 10.26 (− 28.2%), 15.64 (− 11.7%), 45.81 (− 1.8%), and 85.21 (− 6.5%), respectively.

**Conclusions:**

The new technique to obtain images uses a distinct filtering function which considers the mean velocity in the region around each pixel, in turn allowing adjustments according to the characteristics of the phantom inclusions within the ultrasound and optimizing the resulting elastographic images.

## Background

Ultrasound (US) is being used to develop methods to verify tissue elasticity. This technique allows noninvasive and simple diagnosis and monitoring of diseases without altering patients’ examination routine [1].

Ultrasound provides both morphology (images in grayscale) and functional imaging of soft tissues (image stream). Ultrafast technology resources [2] can be used to add a third dimension, pathophysiological formation, by evaluating tissue viscoelasticity. Ultrafast image formation can be used to find transitional shear waves in soft tissues, which spread transversally in this medium. Consequently, reconstructing the image from the shear wave could quantify the mechanical properties of these tissues [3].

Two types of mechanical waves propagate in soft tissue: compression waves and shear waves. Compression waves travel much faster than shear waves in this medium, typically 1540 m/s, in comparison with 10 m/s for shear waves [4]. In other words, the bulk modulus (*K*) for soft tissues is much greater than the shear modulus (*μ*), on an order of 10^6^ [4]. This produces two important consequences: (1) tissue viscoelasticity depends only on the shear modulus, and (2) the difference in propagation speed is so great that shear wave movement can be considered negligible during the propagation time of a compression wave [5]. Since compression waves can propagate in the tissue across a very large range of frequencies (GHz), shear waves are much more strongly affected by the effects of viscosity and attenuation in the tissue [6]. The maximum frequency of shear waves that propagate in human tissues depends on the organ, and typically varies from 500 to 2000 Hz. Consequently, the minimum frame rates needed to correctly show transient waves are in the thousands of Hertz (1000–4000 Hz, considering the Nyquist limit). These frame rates can only be attained using ultrafast architectures [7], and as a result shear wave imaging requires these system models [6].

**Fig. 1:**
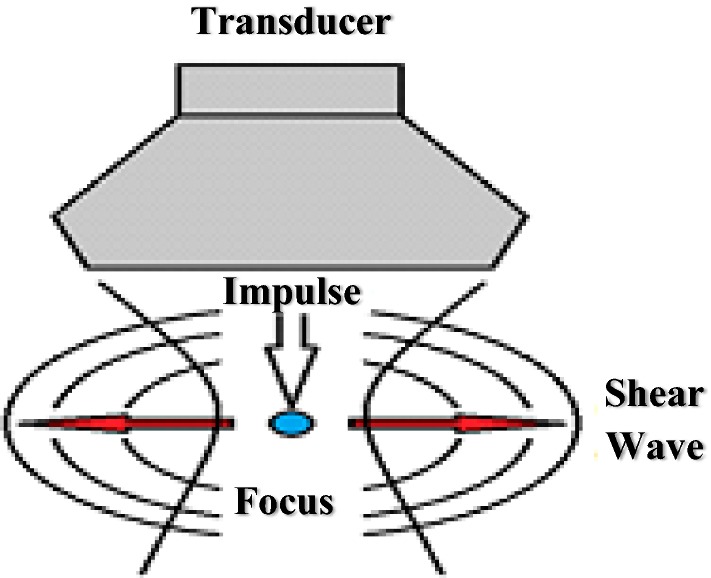
Formation of the shear wave

Transitional shear waves in the body originate from several different types of sources. In 2004, a new imaging mode known as supersonic shear imaging was introduced; the technique is based on induced coupling of radiation force for transitory shear waves and ultrafast imaging (Fig. 1). In this approach, the shear wave is generated and recorded with the same US transducer. This involves inducing a source of shear waves that moves in the body at supersonic speed with the equivalent of a “sonic boom,” creating high-amplitude shear waves in human organs [4–6]. Methods of elastography that vibrate or press the surface of the tissue may have some problems obtaining images of deep regions, because the distortion used to find the elasticity is very weak when it reaches these tissues. However, methods that use acoustic radiation force impulse (ARFI) [8], shear wave elastography imaging (SWEI) [9, 10], supersonic shear imaging (SSI) [11], and shear wave elastography (SWE) [12] may create distortions in deeper tissues, facilitating generation of images in these regions [3–6].

The objective of this study was to use the elastographic technique in developing digital signal processing routines in order to estimate the viscosity and rigidity of structures using a filtering system that calculates the mean values around each pixel. Most of the studies in the literature assess methodologies with variations that focus on different models of transducers or ultrasound equipment [13]. The research that does include adjustment methods to improve image resolution in the inclusion area utilizes a method of calculating velocity [14], a type of inclusion, or biological tissue in vivo [15–18]. These studies refine traditional techniques for obtaining elastographic images such as signal inversion [16] and Butterworth or Kalman filters [17]. This study applies a new methodology which presents an option for adjusting ultrasound images using a median-type filter which is easier to implement. The area can be selected, along with the level of resolution in the elastography, which represents progress in handling shear wave signals in comparison with traditional methods [19–21]. Furthermore, it allows ultrasound technicians the possibility to better interpret the data collected in order to achieve a more precise diagnosis without the use of subjective evaluation methods such as visual interpretation [22].

## Methods

### Relationship between density and shear wave velocity

The relationship between the shear wave velocity and density of the medium was used to derive the elastogram, and considers the sequence of deductions made in the following equations [23–25].

Equation 1 initially represents stress σ, which describes the force applied in a given region by the ratio between force (*F*) (in our specific case, the force provided by the ultrasound transducer) and the area (*A*) in which it is applied [23].

1 $$\sigma = \frac{F}{A}$$

The rheology deformation, which represents the variation of a length in relation to initial length, is calculated using Eq. 2, where *ε* is the rheological deformation, *L* is the final length, and *L*_0_ is the initial length [23].

2 $$\varepsilon = \frac{{L - L_{0} }}{{L_{0} }}$$

Equations 1 and 2 can be used to obtain rigidity or Young’s modulus (*E*). This parameter, which is shown in Eq. 3, describes the longitudinal deformation captured by the US transducer in the form of the RF signal received [23]:

3 $$E = \frac{\sigma }{\varepsilon }$$

Shear stress *τ*, which occurs when an applied force moves surfaces and leaves them at an angle different from the initial angle, is obtained by Eq. 4 [23]:

4 $$\tau = \frac{Fc}{A}$$

where *Fc* is shear force and *A* is the area where force is applied.

Subsequent calculations require shear deformation to be obtained (*γ*), as shown in Eq. 5, by the difference between the length after deformation (*L*) and initial length (*L*_0_) divided by the length after deformation (*L*). This calculation is performed via software through a US signal processing routine and serves as the basis for autocorrelation algorithms [24].

5 $$\gamma = \frac{{L {-} L_{0} }}{L}$$

Young’s modulus measures stiffness in simple extension or compression. There are different ways to deform a material, resulting in different effects on the interatomic forces and consequently different effects on the material [18]. The deformation mode investigated and used in this study was shear stress [6]. Shear stress occurs when an applied force moves parallel surfaces, leaving them at an angle that differs from the initial angle between these two surfaces.

Like Young’s modulus, the shear stress modulus is defined as the ratio between stress and deformation. Equation 6 presents the shear module *G,* which is obtained by the ratio between shear stress and shear deformation [24].

6 $$G = \frac{\tau }{\gamma }$$

Young’s modulus (*E*) and the shear modulus in an isotropic material (*G*) (for which the physical properties are the same regardless of direction) are described by the following equation [24]:

7 $$G = \frac{E}{{2\left( {1 + v} \right)}}$$

where *ν* is the Poisson ratio that quantifies the transverse deformation.

Because of the complexity of describing and analyzing wave propagation in material media, some approximations have been made considering ideal liquids and isotropic and homogeneous solids (acoustic properties are constant in the wave propagation region). According to Cobbold [26], infinite wave propagation through material media is analyzed by introducing two elastic constants known as Lamé coefficients, as shown in Eq. 8:

8 $$2v = \frac{{\lambda_{1} }}{{\left( {\lambda_{1} +\upmu} \right)}}$$

where *λ*_1_ is the first Lamé constant and *µ* is the second.

The first Lamé constant (*λ*) relates transverse deformation to longitudinal stress, and is obtained by Eq. 9. The second Lamé constant or shear modulus in soft tissue relates shear deformation to shear stress (as shown above in Eq. 6) [26]:

9 $$\lambda_{1} = \frac{Ev}{{\left( {1 + v} \right)\left( {1 - 2v} \right)}}$$

The relationship between Young’s modulus and the shear stress modulus in soft tissue is obtained in Eq. 10:

10 $$E = 3\upmu$$

and dynamic viscosity for liquids is represented in the following equation:

11 $$\sigma =\upmu_{2}^{{\frac{de}{dt}}} \to \frac{de}{dt} = \frac{d}{dt}\left( {\frac{{L - L_{0} }}{{L_{0} }}} \right)$$

where *µ*_2_ is the dynamic viscosity, *σ* is stress, and *de*/*dt* is the variation in rheological deformation over time [23].

The models described by Eqs. 3 and 11 express the difference between a solid and a liquid. Forces applied to solids cause deformations, and stress is consequently proportional to deformation; forces applied to liquids or fluids cause outflow, and in this case stress is proportional to the temporal rate of deformation.

In terms of the elastic constants, the equations for longitudinal and transverse waves are as follows:

12 $$Cp = \sqrt {\frac{{\lambda_{1} + 2\upmu}}{\rho }}$$

where *Cp* is the speed of the longitudinal wave and *ρ* is the density of the medium.

The speed of the longitudinal wave, based on transversal deformation and longitudinal stress (as shown in Eq. 9) can also be obtained by:

13 $$Cp = \sqrt {\frac{{E\left( {1 - v} \right)}}{{\rho \left( {1 + v} \right)\left( {1 - 2v} \right)}}}$$

Finally, the velocity of the shear wave *Cs* based on the shear modulus (in Eq. 7) is represented by [26]:

14 $$Cs = \sqrt {\frac{\mu }{\rho }} = \sqrt {\frac{E}{{2\rho \left( {1 + v} \right)}}}$$

According to Lakes [27], typical biomaterials and materials with the characteristics of soft tissue, such as tissue-mimicking elastography phantoms, have a longitudinal wave speed many times greater than the transverse wave speed. In some soft tissue, the longitudinal wave speed is on the order of 1500–1580 m/s, while the transversal velocity is on the order of 0.5–20 m/s. Most living tissue is consequently non-compressible, with the Poisson coefficient ranging from 0.49 to 0.5 [28]. Therefore, the shear wave velocity obtained via US indirectly determines the density of the medium.

Once the ratio of shear wave velocity and the density of the medium are determined, US can be used to generate the SSI shear wave, which generates a wave front perpendicular to the focal point. A processing algorithm is used to calculate the density at different points in the phantom, using US to assess the different speeds of the wave spreading in the lateral positions. The rigidity of the phantom analyzed is determined on a full 2-D map. In post-processing, the filter algorithm is applied to the median of the array surrounding each pixel, which adjusts the elastographic map [26].

**Fig. 2:**
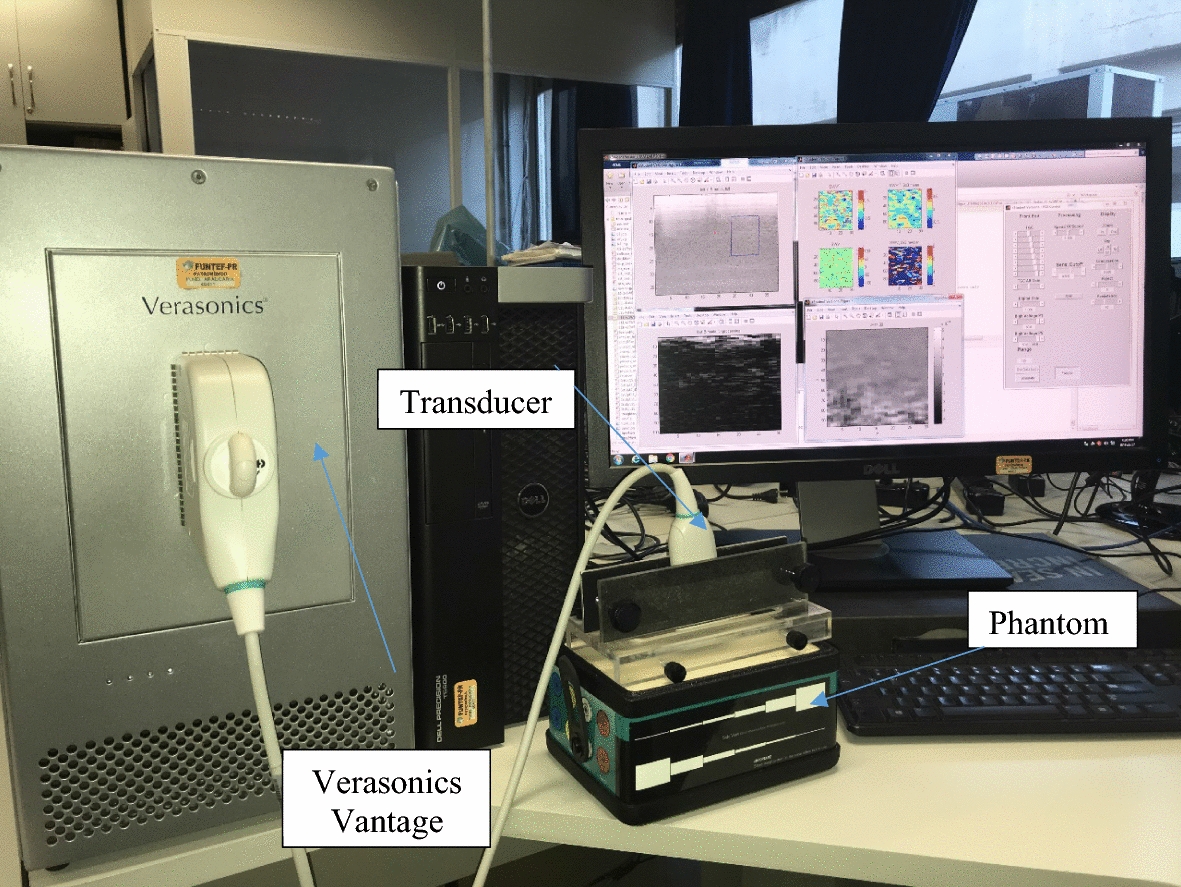
Setup used for data acquisition showing the CIRS 049A elastography phantom [30] and the Verasonics Vantage 128 system and L11-4v model transducer with 128 elements

The impulse sequences were programmed in a Verasonics Vantage 128 US system using a L11-4v model transducer with 128 elements. The research platform consists of two parts: (1) a dedicated hardware system (to transmit and acquire US signals), and (2) a software package with open and proprietary functions running on a computer via MATLAB. The signal acquired is compressed before transferring to the computer; all beam formation and treatment is subsequently done using software. The acquisition modules are responsible for transmitting and receiving the US on each channel. The RF data received are stored in the local memory of these modules, and the data acquisition system is connected to the computer through a PCI cable. Figure 2 shows the setup used for data acquisition.

Table 1 presents the main parameters used in the routine implementation of Verasonics equipment and respective adjustments. These parameters must comply with the sequencing of data processing protocols used in the Verasonics system (shear wave elastography imaging). Selection of the sequencing parameters is described in a system programming script. The data processing line to calculate shear wave speed includes estimating different densities in the phantom and filtering the data to estimate the velocity and subsequently produce an elastogram [29].

**Table 1:** Routine acquisition parameters in MATLAB SWEI structure

Parameter	Values for C11-4v transducer
Impulse frequency	5 MHz
Beam frequency	5 MHz
Impulse duration	1000 cycles of 192 µs
Impulse focus configuration	14.8 mm
Beam focus configuration	Plane wave, fully open
Data channel sampling frequency	20.0 MHz
IQ data beam forming sampling frequency	0.25 λ
Pulse repetition interval	100 µs
Excitation voltage	50 V
Sampling frequency	5000 Hz
Number of transmission channels	128
Number of reception channels	128

The Verasonics system is configured in a MATLAB programming environment. To generate a sequence of images, the user writes a programming script that generates a range of parameters which are loaded on the Verasonics scanner during execution. The objects are defined using MATLAB structures. The parameter base and action sequence are defined when the programming script is executed, and these definitions are filled in and saved in a series of structures; this file can then be loaded into the system by a program manager (VSX) to implement the sequence during execution. The RF data channel can be accessed after the image sequence is completed. If the Verasonics data beamforming is acquired in phase and in quadrature (IQ), the data from the detected image can also be accessed.

The procedures for calibrating the beam position, scanner time, and transducer face heating are based on the routine programming interface specifications of the US equipment manufacturer in order to avoid problems with measuring shear wave speed (SWS) and avoid likely damage to the transducer.

**Fig. 3:**
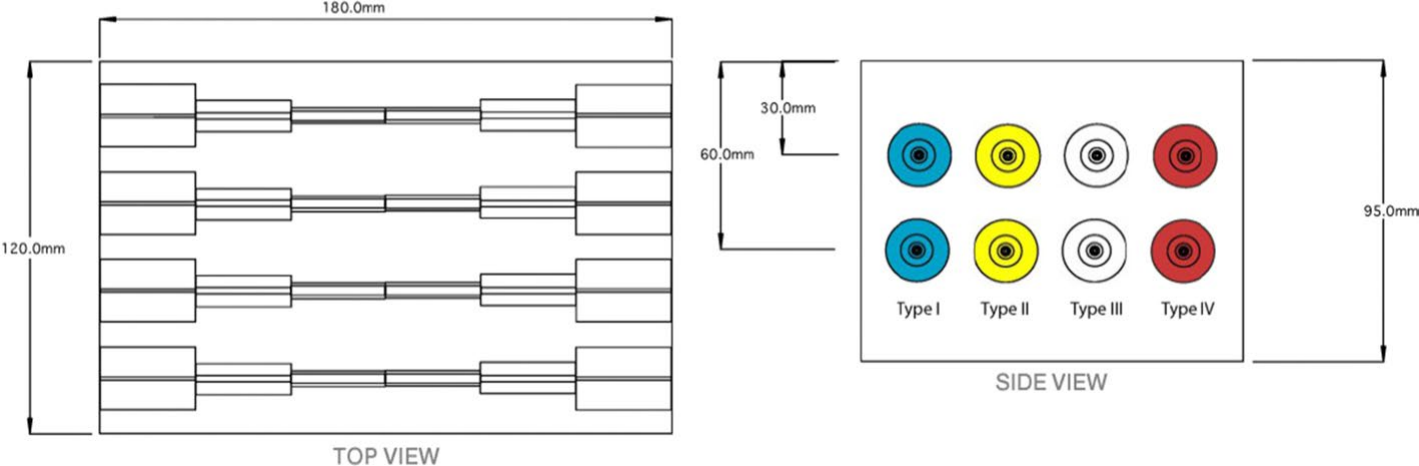
CIRS 049A elastography phantom [30]

For the test sequences, a CIRS model 049A elastography phantom was used (Fig. 3) [30]. This device has eight cylindrical inclusions with gradually decreasing diameters, which are alternately positioned at two different depths with four different types of densities. Values for the elasticity of the 49A phantom models are presented in Table 2. These will be used for comparison of elastographic images obtained after processing.

**Table 2:** Relationship between mold model and characteristic compression. Adapted from 049 CIRS phantom manual [30]

Type of material	Viscoelasticity in kPa
Phantom base	25 ± 6 (24.0%)
I	8 ± 3 (37.5%)
II	14 ± 4 (28.6%)
III	45 ± 8 (17.8%)
IV	80 ± 12 (15.0%)

### Applied algorithm

The phantom was excited with signals configured by the Verasonics device with amplitude of 50 V in plane wave mode; in other words, all the elements of the transducer were active at the same time with rates of 100 frames per second and velocity of 1540 m/s.

**Fig. 4:**
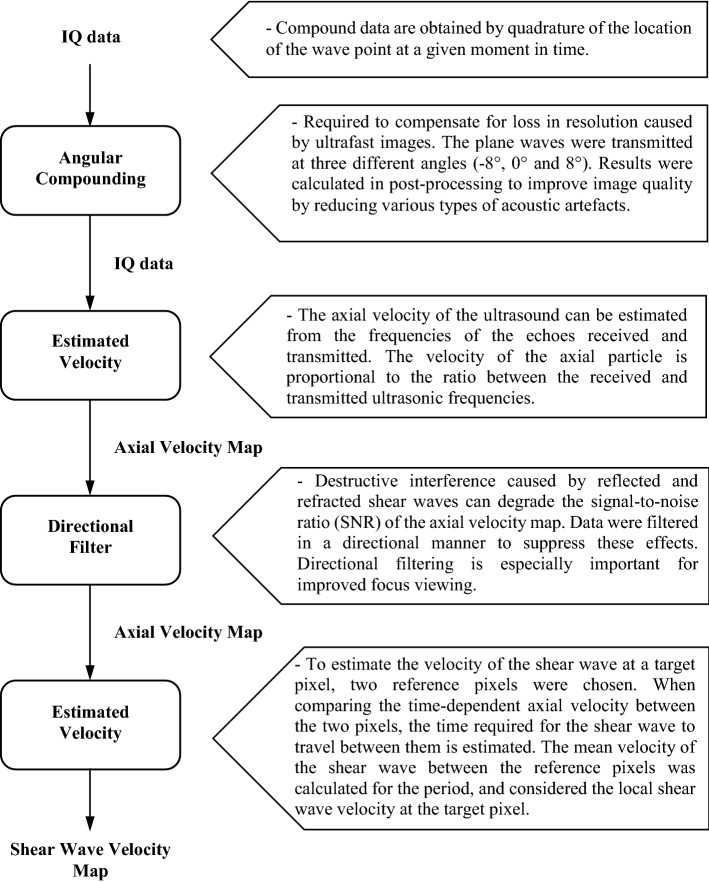
Flowchart of the program sequence for elastogram generation

The event routine is presented in a simplified form in Fig. 4, representing the main stages of the process and the respective results for sequence transition to produce the elastogram.

The IQ data were initially reconstructed by the Verasonics system for each acquisition in B mode. These data were obtained from three different angles (8°, 0°, and − 8°) and then passed through a third-order moving average filter between the frames, generating the angular composition [31]. In this way, each average frame was the result of three average original frames from different angles. The speed of the axial particle is proportional to the Doppler frequency ratio, namely the difference between the frequencies received and transmitted [31].

The resulting IQ signal can be used to estimate the movement of the shear wave. The 2D autocorrelation approach according to Loupas et al. [32] was used to estimate the speed of the local axial particle. Equation 15 shows the final expression for axial speed *v* [32], where *c* is the speed of the ultrasonic waves in the medium, *Ts* is the pulse repetition period, *ts* is the sampling time along the depth, *fc* is the ratio between the wave length of the central frequency wave and RF sample, *M* is the sample depth, *N* is the sample of different frames, *m* is the column number, and *n* is the line number.

15 $$v = \frac{cts}{{4\pi f_{c} Ts}}\tan^{ - 1} \left\{ {\frac{{\mathop \sum \nolimits_{n = 0}^{N - 2} \left[ {\mathop \sum \nolimits_{m = 0}^{M - 1} Q\left( {m,n} \right)\mathop \sum \nolimits_{m = 0}^{M - 1} I\left( {m,n + 1} \right) - \mathop \sum \nolimits_{m = 0}^{M - 1} I\left( {m,n} \right)\mathop \sum \nolimits_{m = 0}^{M - 1} Q\left( {m,n + 1} \right)} \right]}}{{\mathop \sum \nolimits_{n = 0}^{N - 2} \left[ {\mathop \sum \nolimits_{m = 0}^{M - 1} I\left( {m,n} \right)\mathop \sum \nolimits_{m = 0}^{M - 1} I\left( {m,n + 1} \right) + \mathop \sum \nolimits_{m = 0}^{M - 1} Q\left( {m,n} \right)\mathop \sum \nolimits_{m = 0}^{M - 1} Q\left( {m,n + 1} \right)} \right]}}} \right\}$$

As the shear wave propagates in a direction that is perpendicular to its direction of polarization [33], a directed impulse beam is required to produce inclined shear waves to compose the image. The L11-4v linear array transducer was used to produce shear waves from different angles using different parts of the probe. The combined impulse technique, introduced by Song et al. [29] was used to transmit multiple pulse beams simultaneously at different depths of the probe focus. This produces several shear waves of different angles in the different fields of view (FOV) at the same time. The same Verasonics system was used to produce the US beam and to track the movement of the resulting shear wave. The particle speed signals caused by the propagation of shear waves were used as a signal of shear wave movement in this study, calculated from the data for consecutive frames in IQ using a 2-D autocorrelation method [12].

The raw motion signal of the shear wave was calculated using three pixels in the axial spatial dimension, and time with two sampling points in the same direction. Finally, a spatial median filter was used in each frame of the shear wave signal to improve image definition.

### Estimating velocity

The data were initially filtered according to the direction of the waves generated. Discrete Fourier transformation (DFT) for two dimensions was used to convert the data for the frequency domain. A mask was applied to select the quadrants that represent the direction of velocity, obtaining only positive speeds for the shear wave. Finally, inverse Fourier transformation was applied to return the values to the time domain [6].

Two pixels of reference were chosen to estimate the shear wave velocity in a target pixel. In order to compare the axial velocity, which depends on the time between the two pixels of reference, the time required for the shear wave to move between them is estimated. The average speed of the shear wave between the pixels of reference was calculated for each period and considered the speed of the local shear wave at the target pixel [34]. The selected reference pixels had the same depth as the target pixel, and three pixels located laterally to the left and right.

**Fig. 5:**
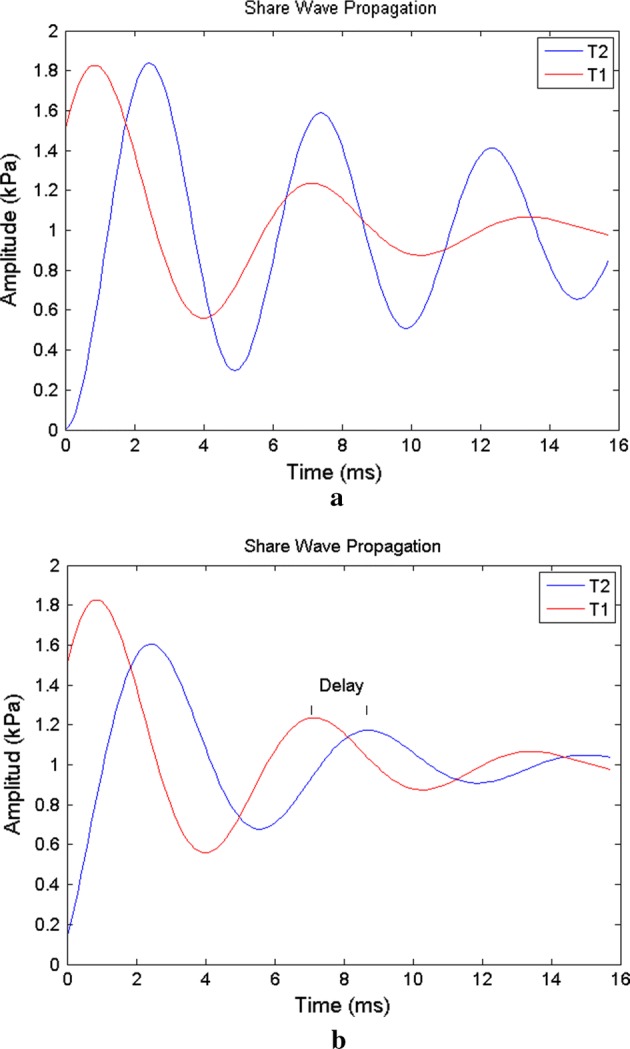
Two shear wave forms from two neighboring pixels **a** before and **b** after directional filtering

The propagation and attenuation of the shear wave can be seen in Fig. 5. Figure 5a shows sample positions of a pixel target in red (T1), together with its reference pixels in blue (T2). A cutting wave that propagates in the lateral direction would first reach one of the reference pixels. Figure 5b shows the axial speeds resulting from two of these pixels (one a reference pixel and the other the target) after directional filtering was applied; this is necessary because of the displacement of the waves, which can move from right to left or left to right. Only one direction matters for determining velocity.

Directional filtering removes interference, leaving the two waves with similar shapes. The cross-correlation can be used to estimate the delay in the arrival times for the shear wave between the two sites [35]. With the array of delay times and the distances traveled, the velocities can finally be obtained by dividing the distance by the respective time.

The frequency response in a Butterworth filter is very flat, without rippling or undulations in the pass band, and approaches zero in the rejected band. For a first-order filter, the response varies by − 6 dB per octave. The magnitude of Butterworth filters drops as a linear function; this type of filter is the only one that maintains the same format for higher orders, although there is a steeper slope in the attenuated band [31]. The Butterworth filter was used in this study to compare the results.

The image processing is based on the elastogram generated to calculate the velocities. Three correction methods were used to show the differences in resolution of the reconstructed images. A Butterworth filter was configured as a low-pass filter, with the following programming function:

$$\left[ {{\text{b}},{\text{a}}} \right] = {\text{butter}}\left( {{\text{n}},{\text{Wn}}} \right);$$

where the response returns the coefficients of the transfer function for a digital low-pass *n*-order Butterworth filter with normalized cutoff frequency *Wn*.

Spectral inversion converts the response to an impulse as the pass band becomes the block band, and vice versa. In other words, this procedure transforms a low-pass filter into a high-pass one, and a high-pass filter into a low-pass filter. Since the low-frequency components were subtracted from the original signal, only the high-frequency components remain [31]. Spectral inversion was also utilized in this study to compare the results.

The filtered data were applied to the inverted signal method, where the result of the velocity matrix is calculated using the value of each inverted cell via the following function:

$${\text{Y}} = {\text{inv}}\left( {\text{X}} \right);$$

where *X* is the estimated shear wave speed after applying the Butterworth filter.

Finally, the proposed filtering algorithm for the medium is applied using a sub-array. This function allows the array size to be adjusted to determine the median value that best fits the image (in this specific case, 3 × 3). Median filtering with the *medfilt2* function follows the routine in the attached file. The complete calculations and images generated are presented for comparison.

## Results

**Fig. 6:**
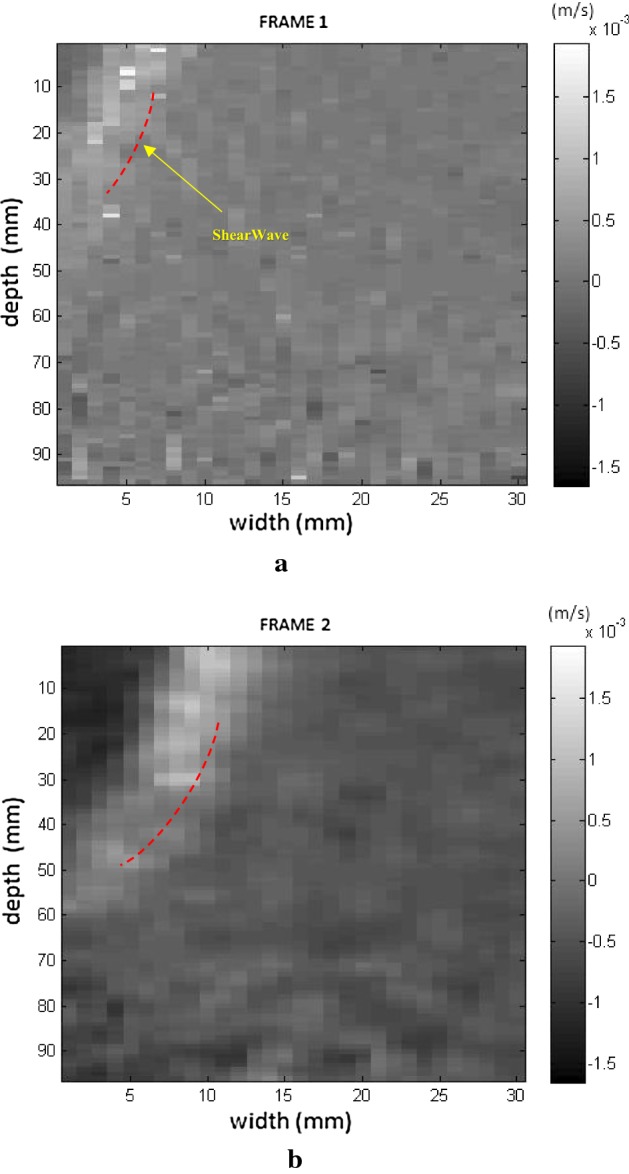
Screens showing the displacement of the supersonic shear wave cone (dotted line) starting on the left (**a**), crossing the phantom (**b**) to the right and respective velocity in the time sequence

**Fig. 7:**
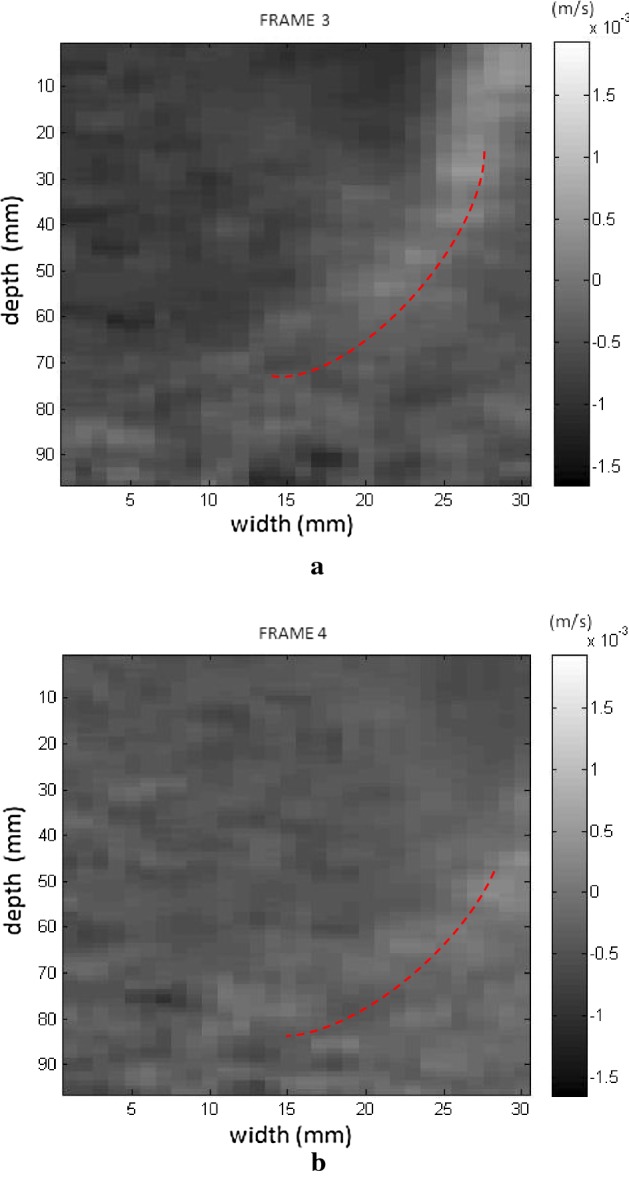
Screens showing the displacement of the supersonic shear wave cone (dotted line) crossing the phantom (**a**) to the right (**b**), along the FOV, and respective velocity in the time sequence

Initially, images were obtained which confirmed the displacement of the supersonic shear wave in the phantom in two directions. In the experimental test, displacement occurred in a linear manner in real time to demonstrate the process of generating shear waves. The cone of the selected supersonic wave travels across the region of observation from left to right, as seen in the sequence in Figs. 6, 7 where 4 frames were captured progressively to demonstrate the movement of the wave.

**Fig. 8:**
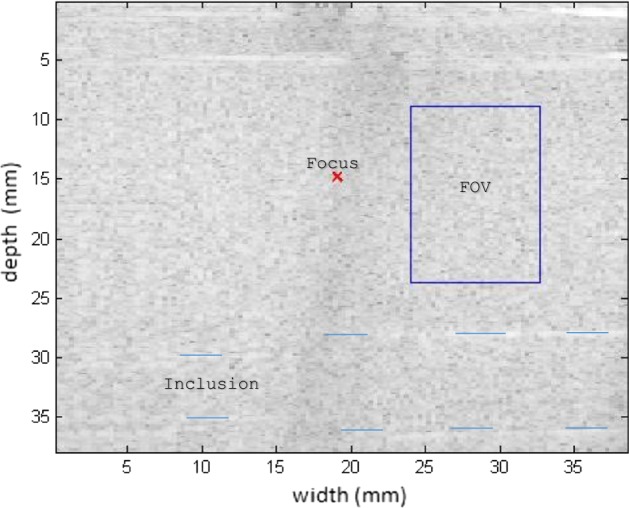
Screen in B mode for the ROI in a homogeneous area in the 049 CIRS phantom; note the inclusion positioned below as a reference

Measurements were taken in the 049 CIRS phantom, to compare the levels of viscoelasticities cited in Table 1. The measurements were also taken at different positions to emphasize the variation with respect to the medium. For the first measurement of the phantom, a homogeneous region (without any inclusions) was chosen for testing. The spatial region of interest (ROI) selected is above the phantom inclusions (blue line), as seen in Fig. 8 in B mode. The elastographic maps were generated after processing the RF signals.

**Fig. 9:**
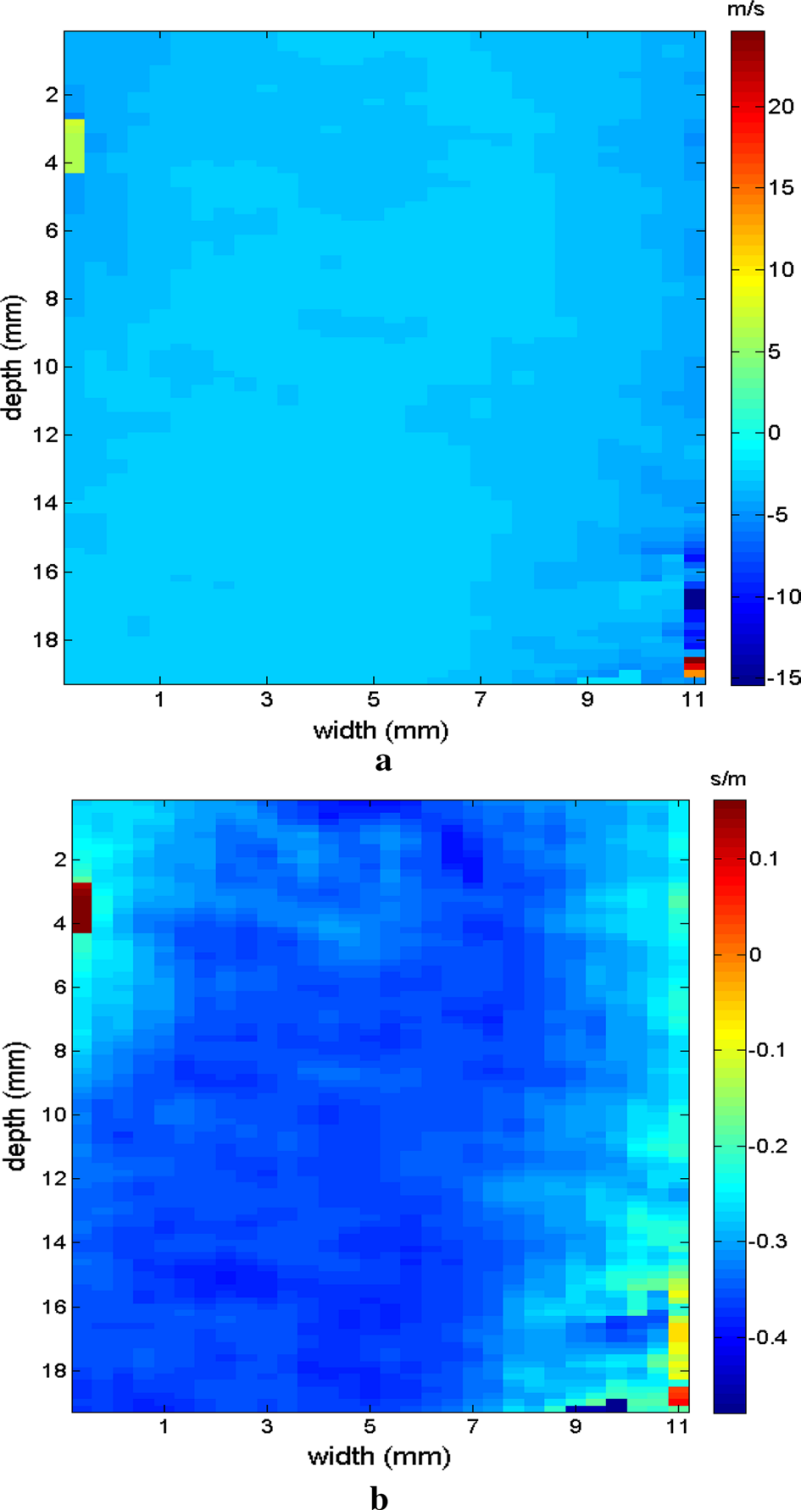
Elastograms positioned in the homogeneous area of the phantom referring to Fig. 8, using the following methods: **a** default elastogram, **b** low-pass filter

**Fig. 10:**
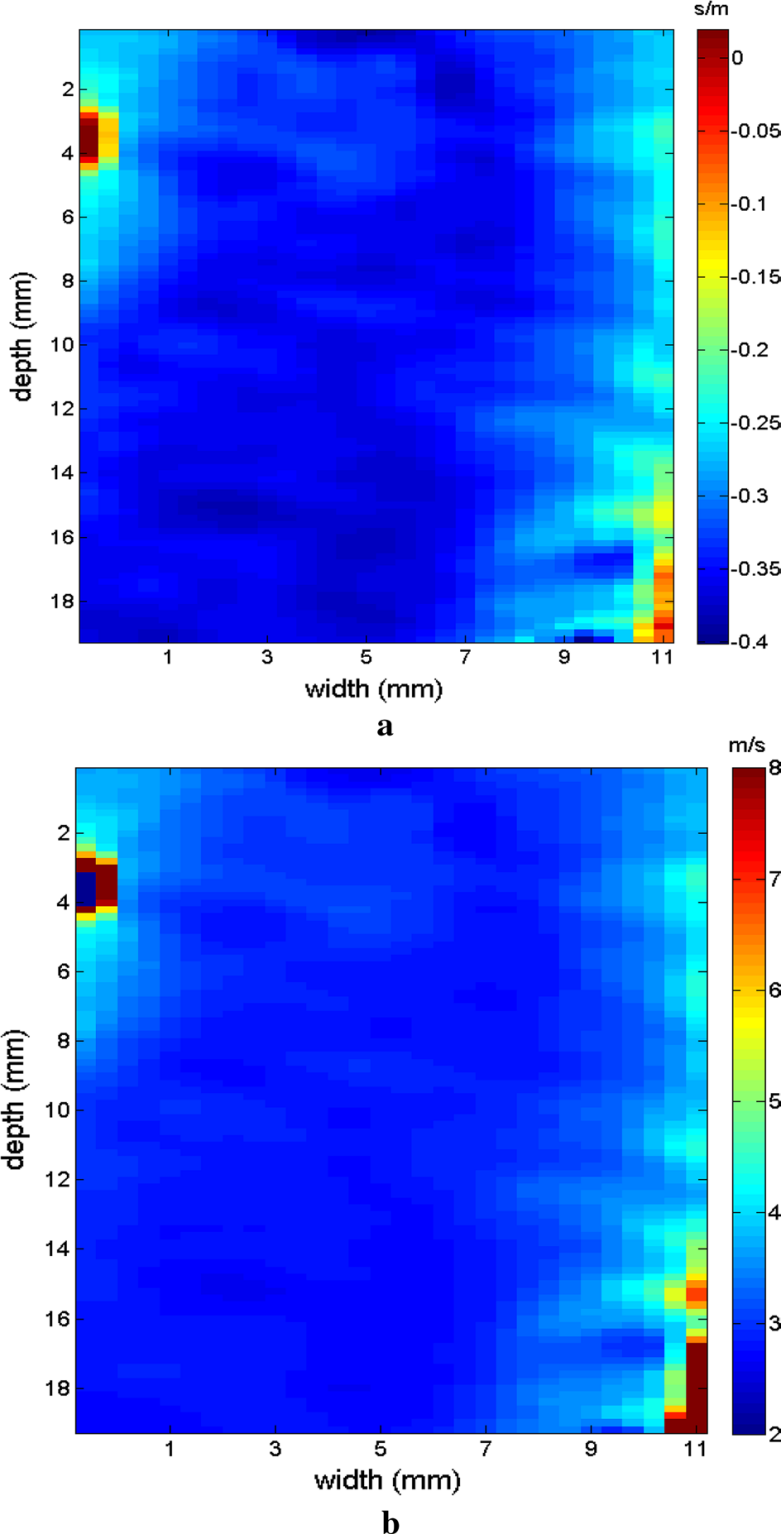
Elastograms positioned in the homogeneous area of the phantom referring to Fig. 8, using the following methods: **a** signal inversion, and **b** median filter

Figures 9, 10 shows the elastograms positioned in the homogeneous area of the phantom referring to Fig. 8 (ROI without inclusions), using the following methods: (a) default elastogram, (b) low-pass filter, (c) signal inversion, and (d) median filter. Note that there is no distortion in the shear wave velocity, and consequently the elastographic map remains uniform in the region of observation. The column to the right of the elastograms presents viscoelasticity values proportional to the shear wave velocity in m/s. These characteristics demonstrate the effectiveness of this method and serve as a reference for subsequent measurements and calculations.

Next, the elastogram was produced using the traditional shear wave velocity technique (Fig. 9a). The resolution of this image was compared to identify the inclusion and differences in each method. First, a default low-pass Butterworth filter was applied (Fig. 9b). Next, an image correction method with signal inversion was used (Fig. 10a), followed by the proposed median spatial filter set to a 3 × 3 pixel array used in each frame of the shear wave signal (Fig. 10b).

The proposed filter is based on MATLAB’s medfilt2 function. This function can perform median filtering of the data array for shear wave velocity in two dimensions. Each output pixel contains the average value around the corresponding pixel of the input image, and can also control the limits of this array and assign the values for the edges of the image.

The filter uses an algorithm with a linear function to perform the smoothing procedure. The results of the processing can be analyzed in the Fourier domain or in a frequency domain. The response of a linear spatial smoothing filter is the average of the pixels contained in the vicinity of the filtering mask. These filters are sometimes called mean filters, where the size of the mask array determines the degree of detail loss and the degree of smoothing. The median filter scans the image and calculated a region around each point in the image, calculating the median values in the region and replacing the value of the point with this median value. The smoothing filter eliminates noise while preserving the contour of the image.

**Fig. 11:**
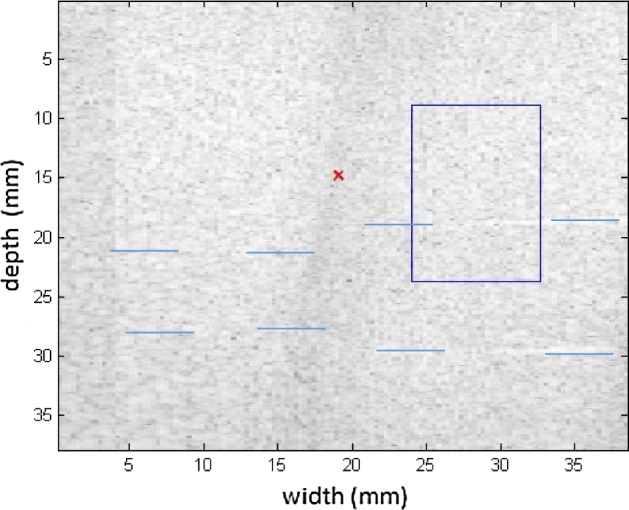
Screen in B mode showing type I inclusion in the 049A CIRS phantom, with half of the area of the ROI positioned in the homogeneous area

The next step was to obtain the elastographic maps for each internal model of the phantom. To differentiate the analyses, the measuring area was altered slightly by displacing the ROI. This shows how the relationship between the area of inclusion with respect to the medium is modified to observe the changes in results. Figure 11 presents the type I model and the relationship between the medium and the elastographic mold is close to 50%. Here the inconclusive region appears on the left, serving as a reference for the focal point of the shear wave’s origin.

In the other measurements, the type of model and the proportionality ratio with the elastographic phantom medium were altered. Measurements were progressively taken of the molds with greater response to pressure and changes in the area of observation where the phantom 049A CIRS was included.

**Fig. 12:**
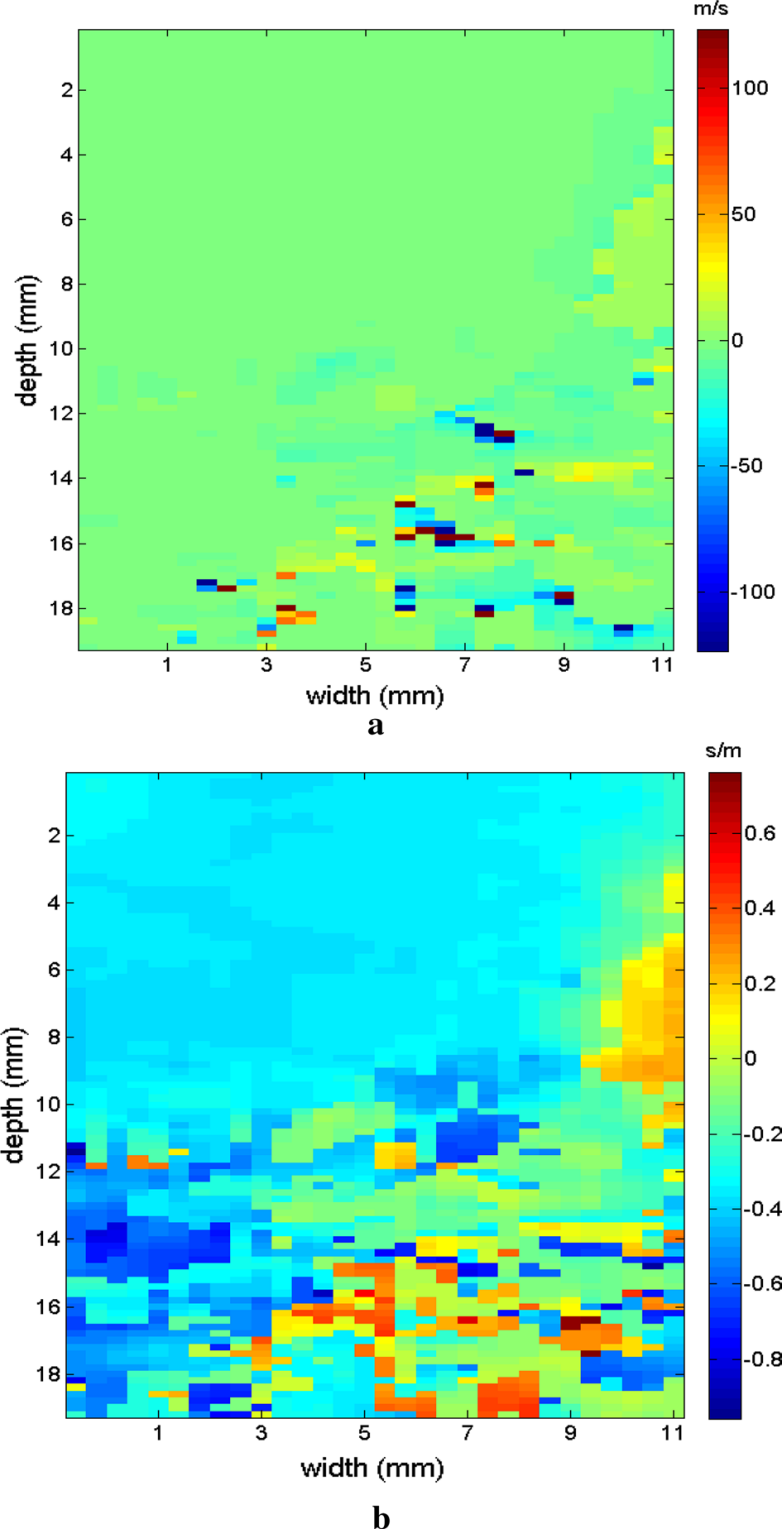
Elastograms referring to Fig. 11 with the ROI showing half of its area in the homogeneous portion (light blue), using the following methods: **a** default elastogram, **b** low-pass filter

**Fig. 13:**
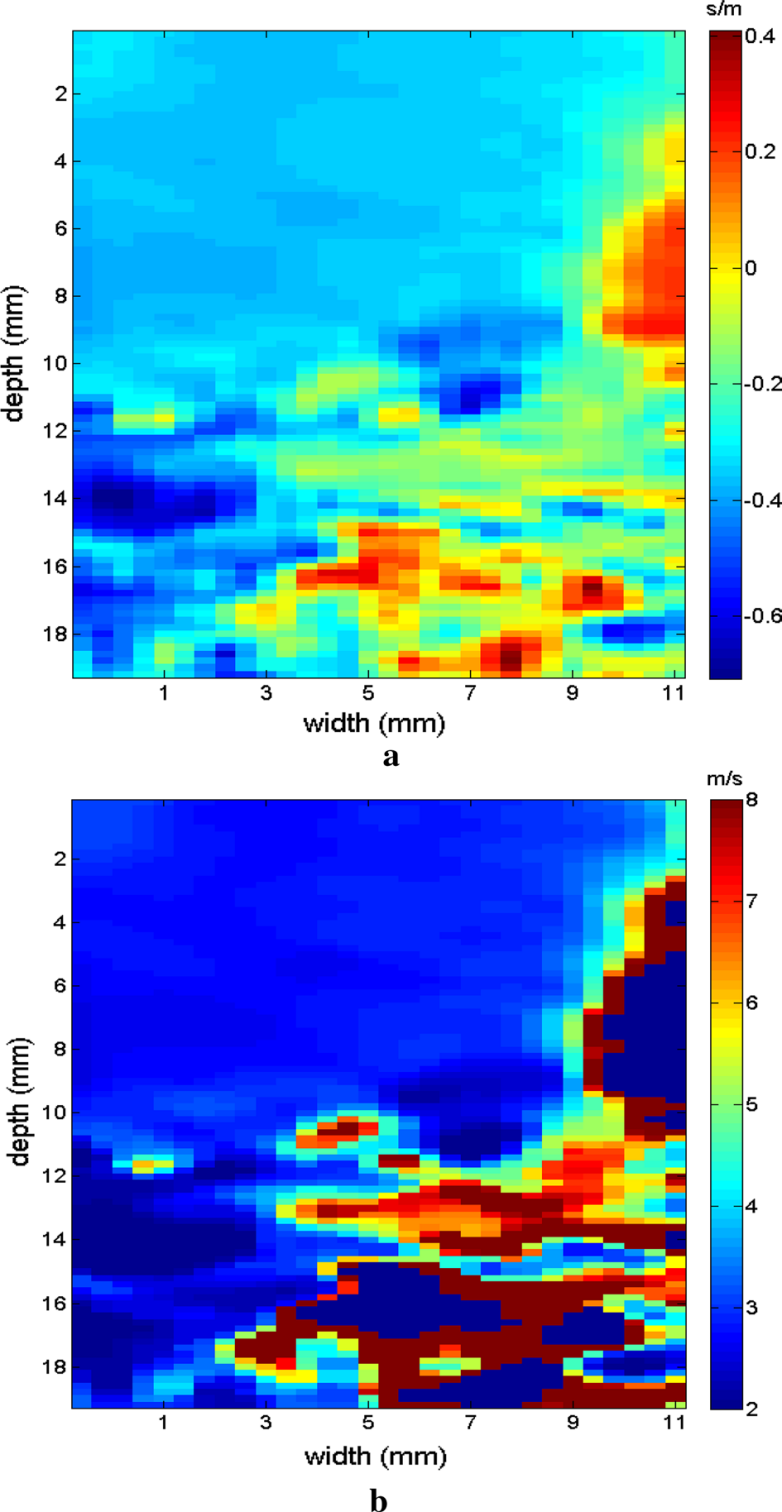
Elastograms referring to Fig. 11 with the ROI showing half of its area in the homogeneous portion (light blue), using the following methods: **a** signal inversion, and **b** median filter

The region of the inclusion presents a variation in the peak axial velocity of the shear wave, and it is consequently possible to distinguish the area in the maps in Figs. 12, 13. The values show variations due to the difference in elasticity, which is calculated by the interpolation used which analyzes the shear wave velocity through the pixels as a function of the fixed distance for determining the variation in velocity. The homogeneous area remains constant, regardless of the method used for interpolation or the filter used. For all methods, the inconclusive region continues to be displayed in the upper left corner of Figs. 12, 13 as a reference for the origin of the shear wave. The filter settings and reference distance for interpolation remained constant in all measurements from the phantom to facilitate interpretation and comparison of the elastograms.

**Fig. 14:**
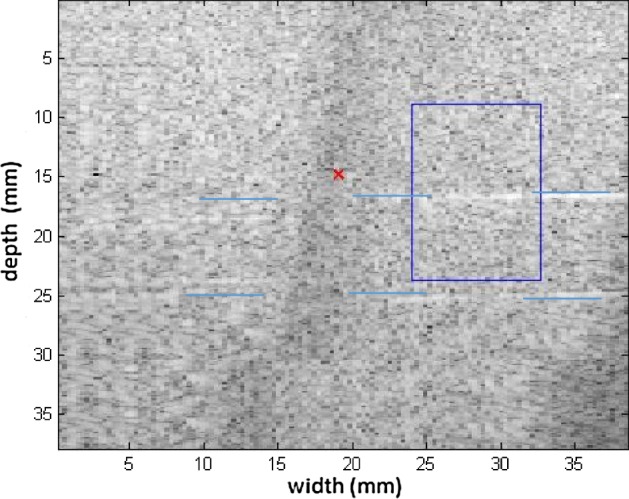
Screen in B mode showing type II inclusion in the 049A CIRS phantom, with the ROI altering the homogeneous area for clearer analysis of the elastogram

**Fig. 15:**
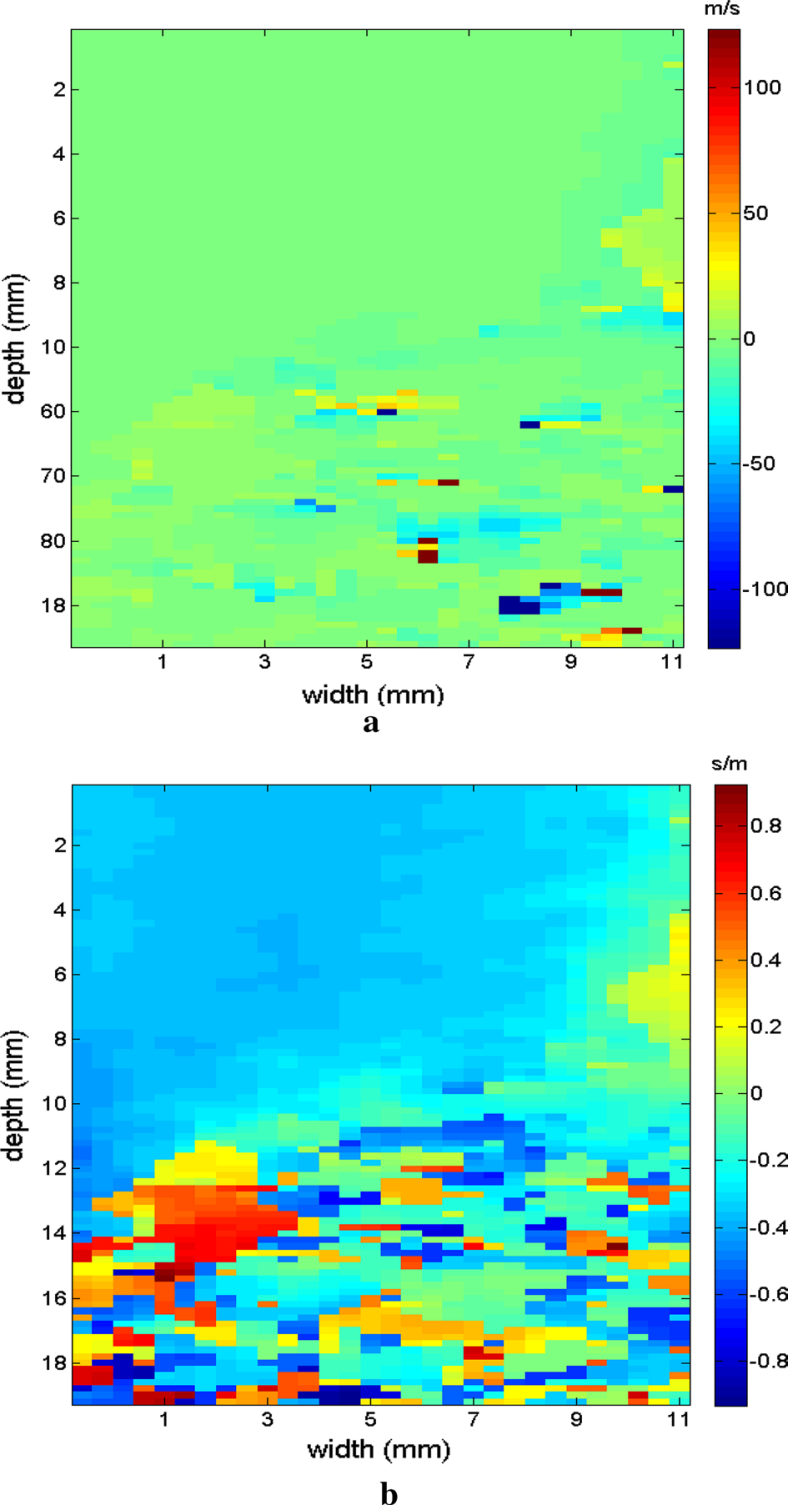
Elastograms referring to Fig. 14, which comparatively show the increase in the area of the inclusion, emphasizing the results of the elastogram, using the following methods: **a** default elastogram, **b** low-pass filter

**Fig. 16:**
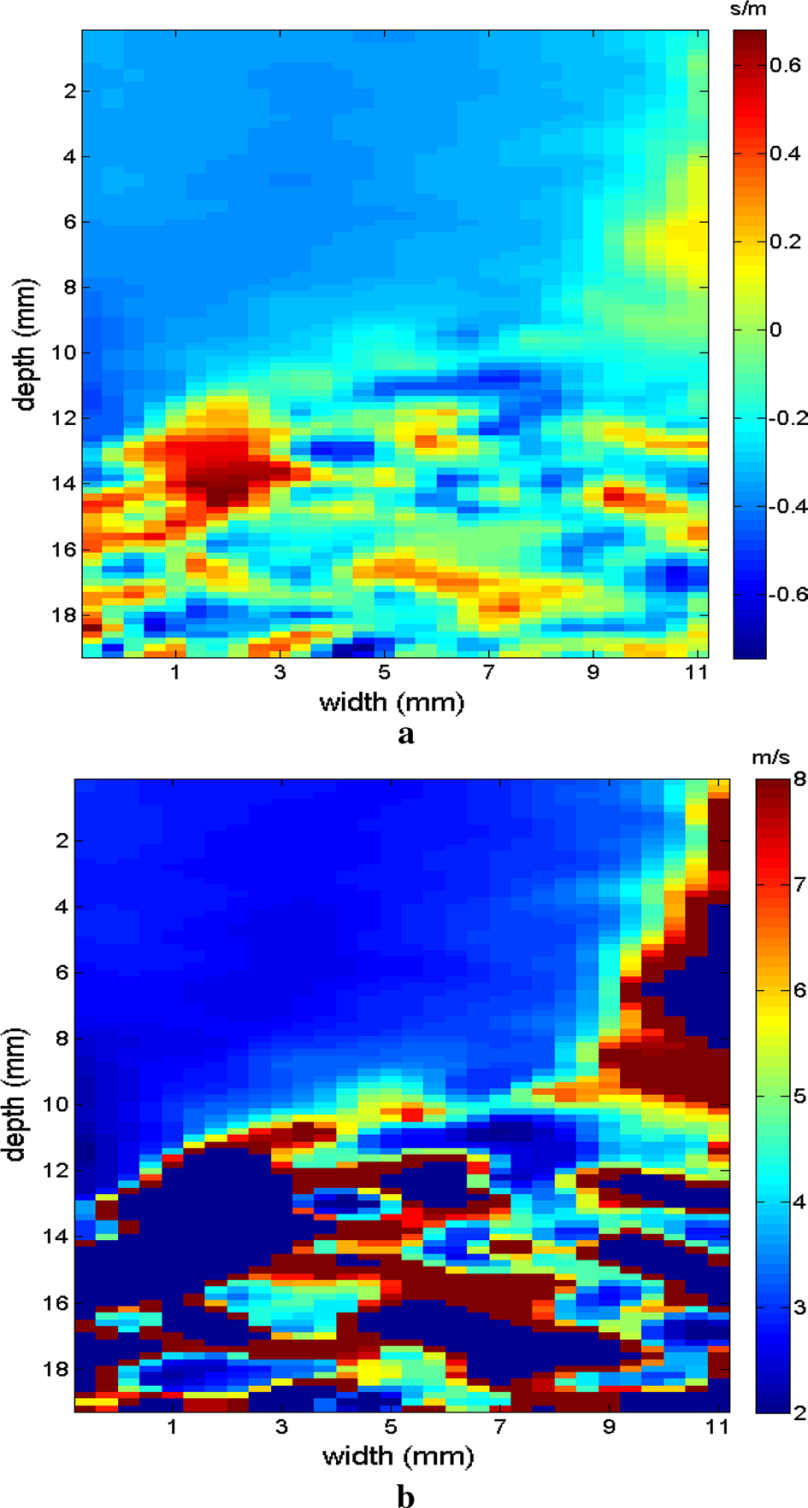
Elastograms referring to Fig. 14, which comparatively show the increase in the area of the inclusion, emphasizing the results of the elastogram, using the following methods: **a** signal inversion, and **b** median filter

**Fig. 17:**
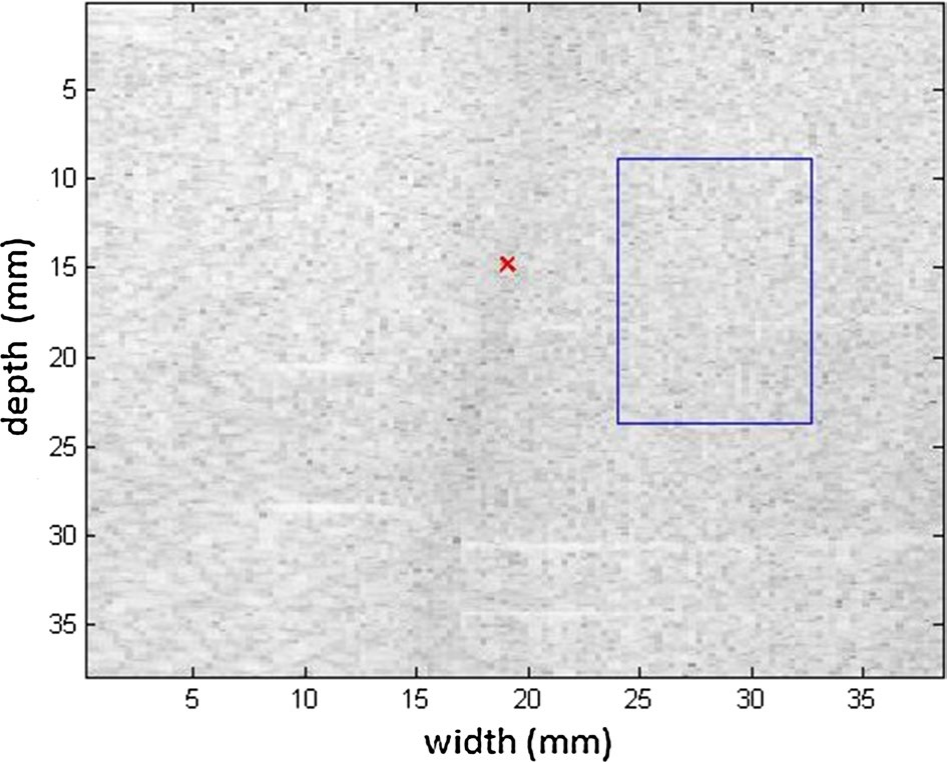
Screen in B mode showing type III inclusion in the 049A CIRS phantom, emphasizing the progressive alteration of the area of inclusion in order to differentiate the results, demonstrating the efficacy of the elastogram

**Fig. 18:**
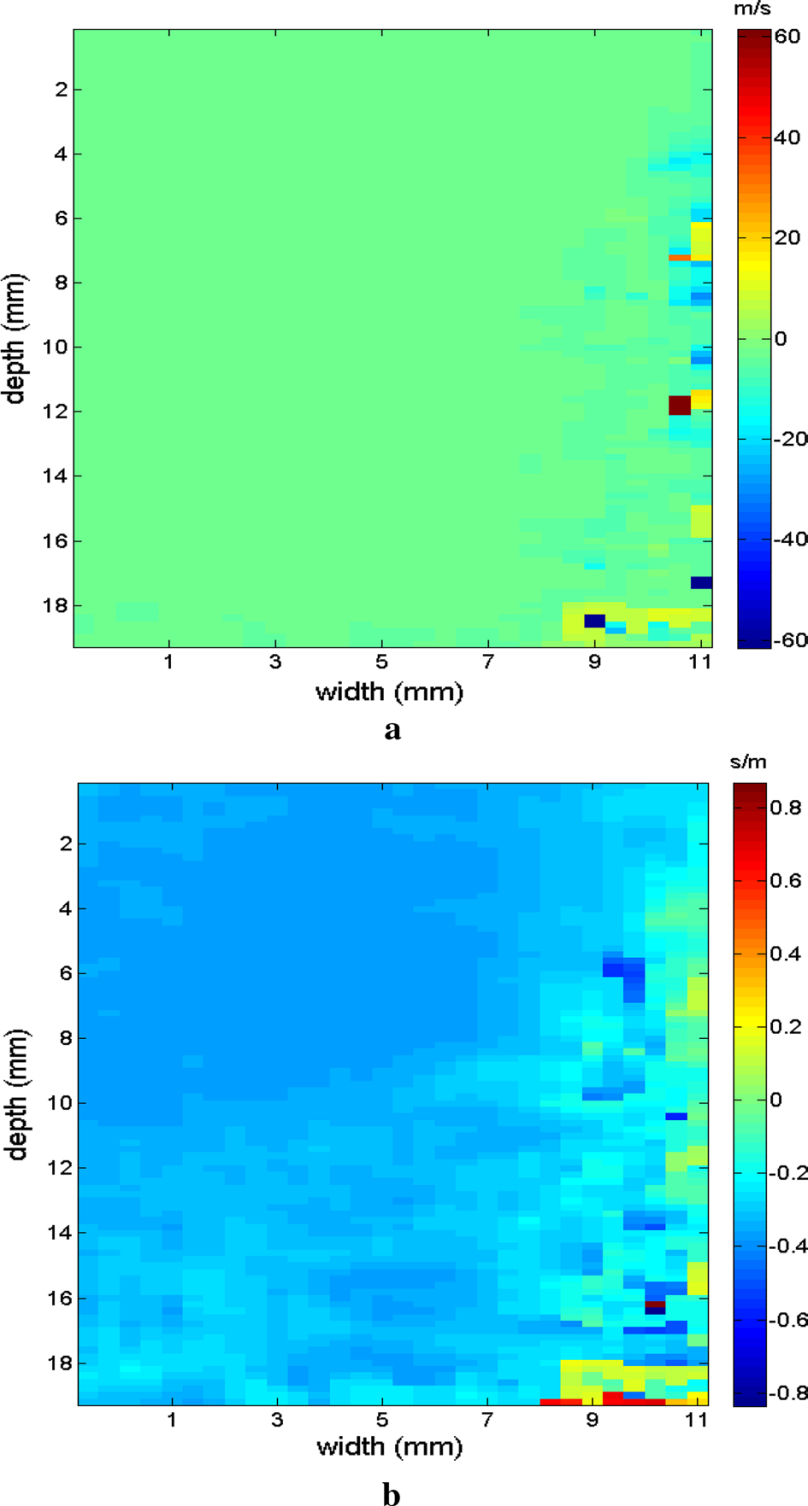
Elastograms referring to Fig. 17, which features the area of inclusion in obtaining the elastogram, using the following methods: **a** default elastogram, **b** low-pass filter

**Fig. 19:**
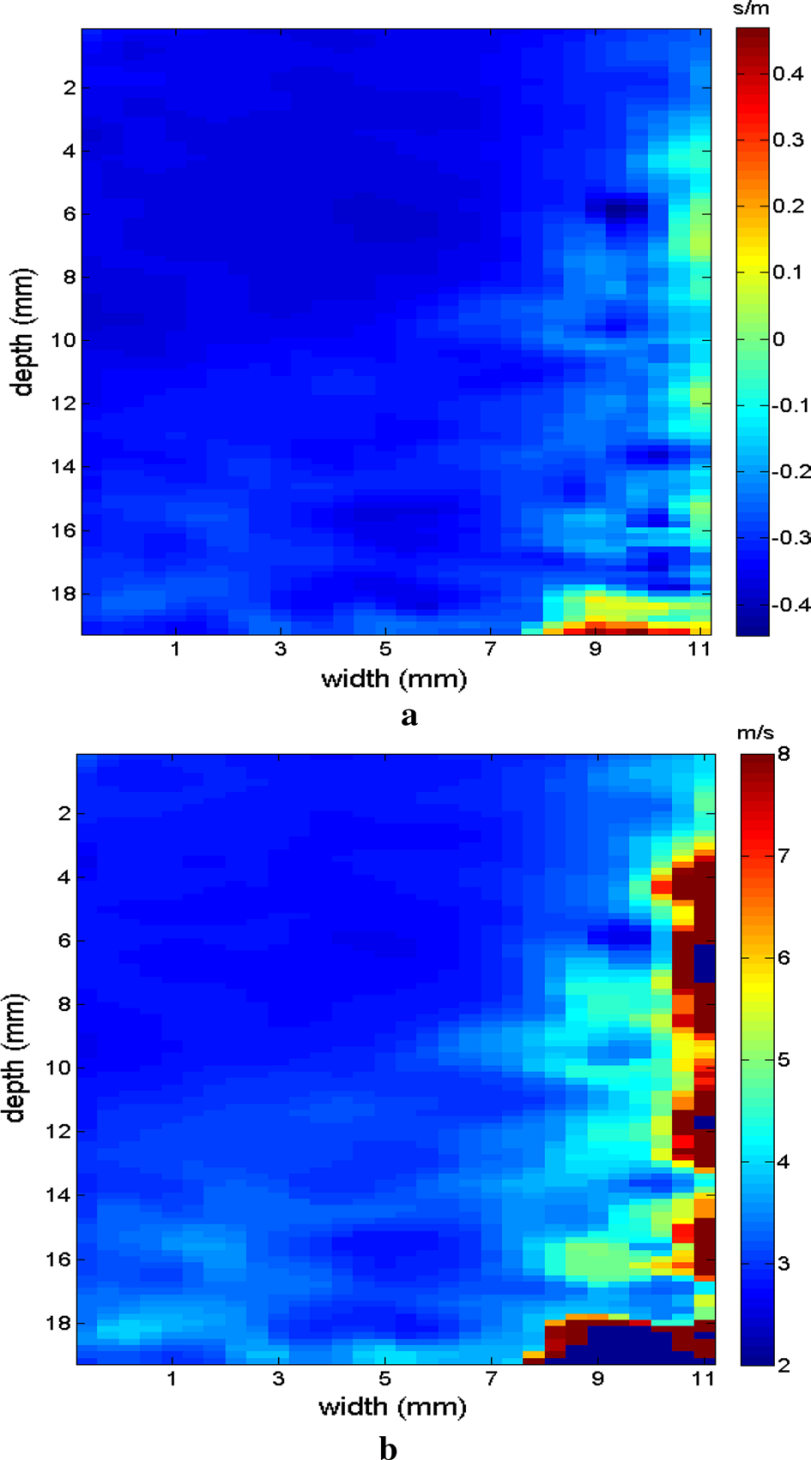
Elastograms referring to Fig. 17, which features the area of inclusion in obtaining the elastogram, using the following methods: **a** signal inversion, and **b** median filter

**Fig. 20:**
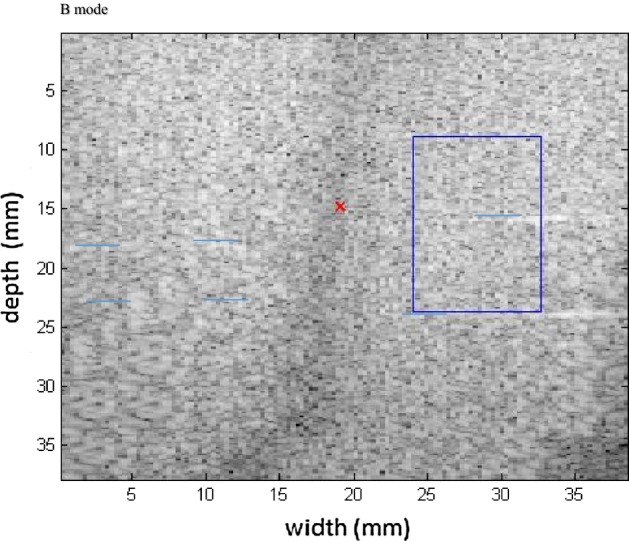
Screen in B mode showing type IV inclusion in the 049A CIRS phantom

**Fig. 21:**
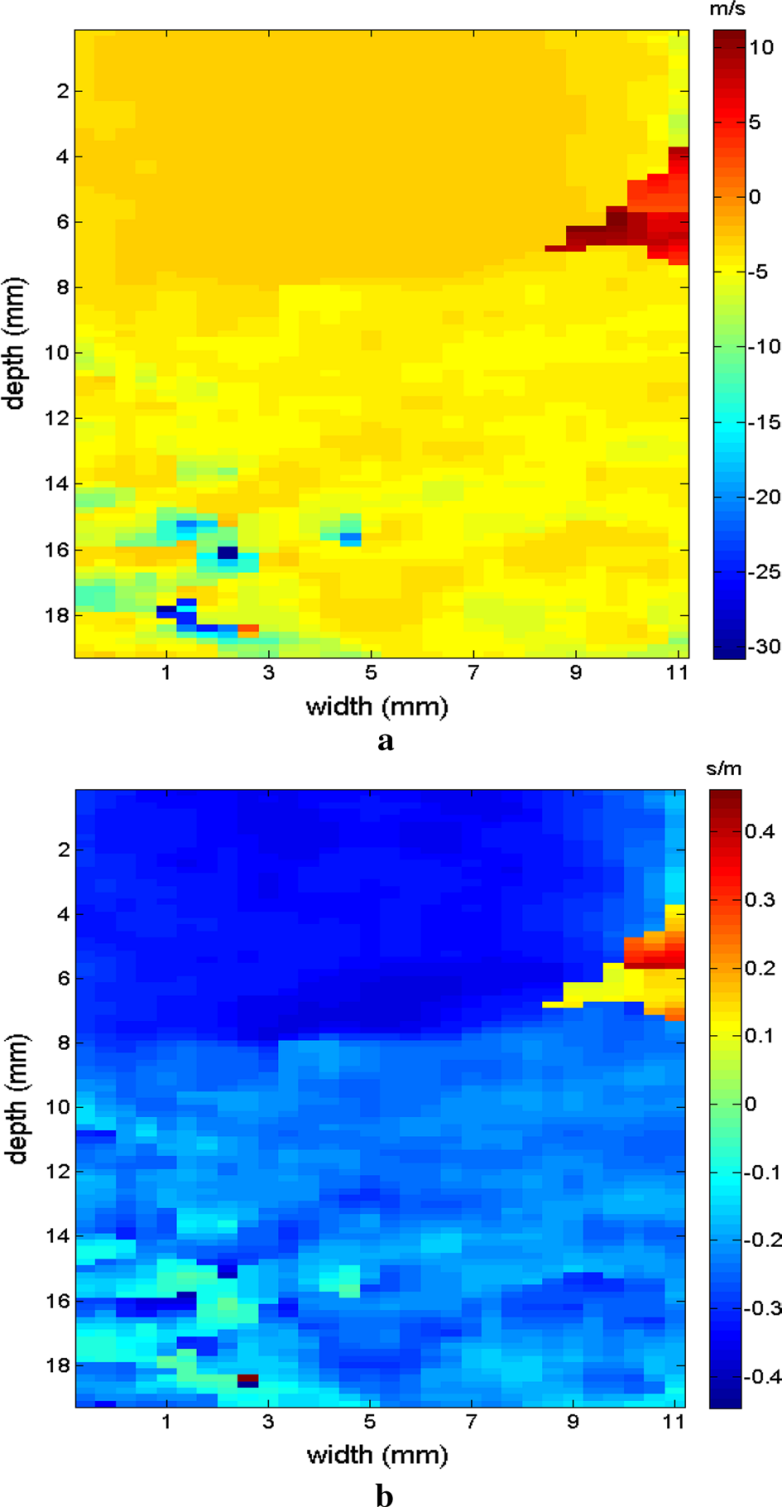
Elastograms referring to Fig. 20; note that in addition to changes in the area of the inclusion, the value also changes proportional to the viscosity of the types of phantoms used, using the following methods: **a** default elastogram, **b** low-pass filter

**Fig. 22:**
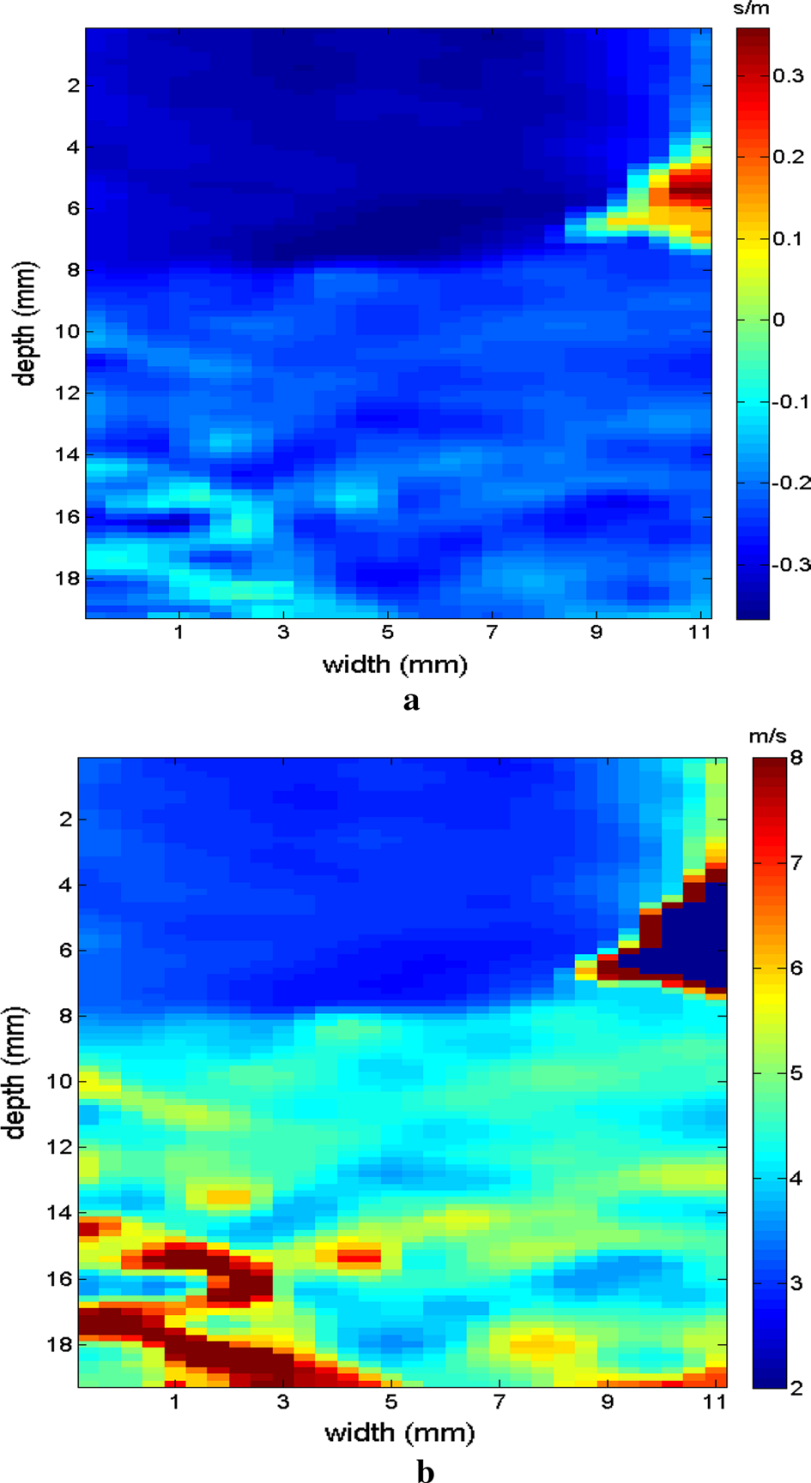
Elastograms referring to Fig. 20; note that in addition to changes in the area of the inclusion, the value also changes proportional to the viscosity of the types of phantoms used, using the following methods: **a** signal inversion, and **b** median filter

Next, the measurements were taken for the other molds of the phantom. Type II is presented in Fig. 14 in B mode, with the inclusion highlighted, followed by the respective elastographic maps in Figs. 15, 16. Type III is presented in Fig. 17 in B mode and the respective elastographic maps in Figs. 18, 19. Type IV is presented in Fig. 20 in B mode and the respective elastographic maps in Figs. 21, 22.

After the graphics were obtained, the velocities were assessed for each inclusion model with respect to the values supplied by the manufacturer, considering the variation in shear wave velocity within the phantom, which were used to calculate the elasticity using the proposed method. The values provided by the phantom manufacturer and the results obtained are presented in Table 3.

**Table 3:** Comparison of obtained results for elasticity (kPa) on the elastography phantom

Type of material	Expected elasticity (kPa)	Measured elasticity (kPa)	Mean error (%)
Phantom base	25 ± 6	29.18 ± 10	− 16.7
I	8 ± 3	10.26 ± 5	− 28.2
II	14 ± 4	15.64 ± 9	− 11.7
III	45 ± 8	45.81 ± 11	− 1.8
IV	80 ± 12	85.21 ± 13	− 6.5

Comparison of the results obtained from the Butterworth filter, signal inversion, and the proposed median filter processing methods using the elastography phantom appear in Table 4.

**Table 4:** Comparison of the results for the Butterworth filter, signal inversion and the proposed median filter processing methods using the elastography phantom

Type of material	Error for Butterworth filter method (%)	Error for signal inversion method (%)	Error for the proposed median filter (%)
Phantom base	16.9	16.8	16.7
I	35.2	31.4	28.2
II	12.5	12.2	11.7
III	4.2	3.3	1.8
IV	9.8	8.3	6.5

## Discussion

Many approaches have been used to estimate shear wave velocity, such as inversion of the second-order wave equation and inversion of the Helmholtz equation [12]. Shear wave speed is estimated from the equation of motion for waves in media; these methods only use waves that are present in the tissue. Both approaches invoke second-order derivatives, which are difficult to estimate due to the low signal-to-noise ratio (SNR) which is inherent in the data [12]. To overcome this limitation, time-of-flight (TOF) approaches based on estimates of shear wave velocity have been introduced [36].

These approaches can be divided into various forms of cross-correlation (CC) and time-to-peak methods (TTP). The 2D approach to calculating TOF speed based on CC was developed and implemented to estimate shear wave velocity from any direction of the propagating wave [37, 38]. Multiple cross-correlations were run along the direction of wave propagation to provide an average estimate for the shear wave speed. The TTP method reduces the problem to a first-order spatial differentiation along a 2D map representing the maximum time of arrival of the wave form [12]. However, these methods require computational time and sophisticated hardware.

Implementing the data processing routine described in this study not only visualized shear wave action, it improved perception of the effects of these waves through the images compared to methods other authors have used to obtain elastographic images [12, 36, 37].

In relation to Engel’s estimation method [12], the inclusion evaluation array could be adjusted, which smoothed the contours. And median filtering produced better image quality than the traditional method developed by Tanter [36], despite greater computational costs. The method presented by Song et al. [39] is one of the scientific bases used in this work. The condition used to differentiate the technique in obtaining the elastogram was the medfilt2 function, which expands the possibilities for image correction but comes with the disadvantage of not eliminating the inconclusive region, requiring redirection of the ROI to remove it from the elastogram.

Another major advance was the ability to implement all the data processing on the Verasonics research platform, combined with the fact that improving the generation of shear waves without relying on external sources such as other transducers, for example, provided a better solution than other methods, such as those presented by Zhao et al. [38].

The support offered by the manufacturer focuses on routines which are already included in the equipment. However, researchers in the area have been conducting innovative studies, since this equipment allows implementation of open hardware routines and diversified research possibilities including Doppler, synchronization of multiple systems, RF filters, and 2D autocorrelation algorithms [32].

**Fig. 23:**
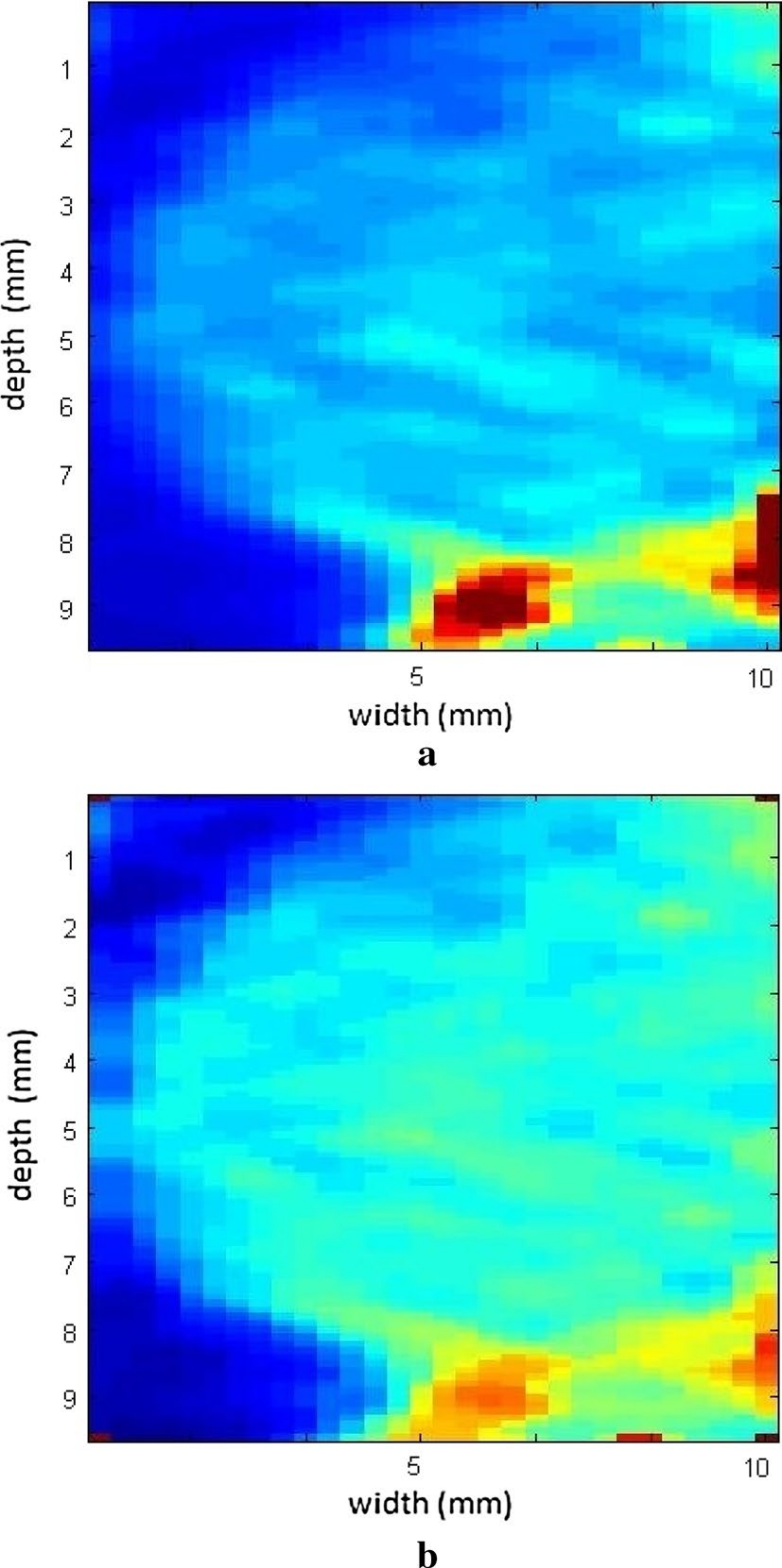
Elastograms without the median filter (**a**) and using the filter (**b**) in a 059 CIRS breast phantom

The filter applied using the medfilt2 function was typified by more uniform representation of the regions where the phantom was included, compared with the methods proposed by Nordenfur [31], Lu et al. [17, 40], and Song [35], which were the foundations of this work. Figure 23a shows that the region of inclusion in a breast phantom (059 CIRS) can be best delimited by color contrast without damaging the interpretation of the viscosity value to be measured, compared with the traditional filtering method used by Carlsen et al. [41], (Fig. 23b).

Another important feature is the ability to adjust the pixel array region (from 3 × 3) to compare the standardized median value during the process of obtaining the images, which may be appropriate depending on the size of the inclusion to be detected. The ROI can be resized in proportion to the size of the inclusion for more uniform representation; in other words, if the inclusion is larger than the initial array, a new array covering this region can be resized to improve visualization for the operator, making the area of analysis more linear and facilitating interpretation of the real dimensions of the inclusion.

**Fig. 24:**
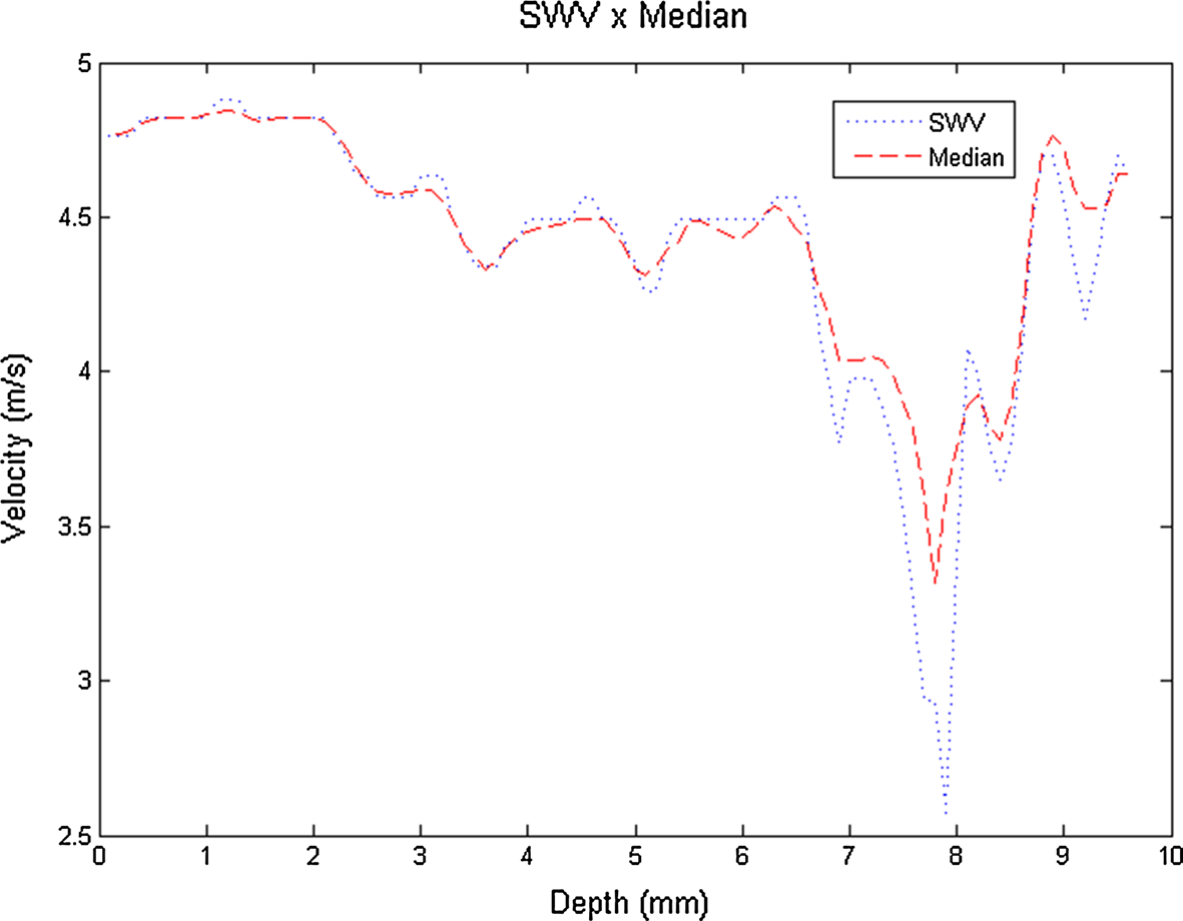
Comparison of the column of images generated from the original elastogram (blue) and the same column using the median filter (red)

Analysis of the results presented in Fig. 24 shows the linearization of values for the regions of the four different types of inclusion in the phantom, as well as the smoothing of contours, improving interpretation of the dimensions of these regions. In quantitative terms, the processing of the signals acquired was proven by the respective results presented in Table 3, as a function of the elastograms obtained.

The methodology for obtaining elastographic images depends on the difference in velocity between the pixels which are next in line to calculate viscoelasticity. However, the adjacent pixels above and below may have slightly different values depending on the quality of the RF data acquired and the choice of the distance between them which is used for calculation. Median array filtering tries to correct these errors, and the medfilt2 function permits more sensitive adjustment to assist in determining the result of exam images. Figure 24 shows the effect of this processing by selecting a column of images generated for the method with and without filtering, which demonstrates the smoothing effect, decreasing the variation in velocity values for the shear wave in the phantom, particularly when the wave meets the inclusion.

Analysis of the results shows that the difference was elevated for type I and II inclusions; however, considering that the standard deviation provided by the manufacturer exceeds 28% (as shown in Table 2), the values are acceptable. For type III and IV inclusions, the percentage error was low (bellow 7.0%).

The images obtained with the proposed median filter method clearly show better resolution when compared with images obtained using the methods in the literature (Butterworth filter and signal inversion) (see Figs. 12, 13, 15, 16, 18, 19, 21 and 22). Table 4 shows the quantitative analysis of the results using the Butterworth filter [31], signal inversion [31], and the proposed median filter method. The average error is within the manufacturer’s margin, and is always lower than the error for other methods, which corroborates the efficacy of the median filter method.

## Conclusions

This article is relevant because of the experimental tests performed to validate the proposed method based on commercial phantoms and analysis of its performance in relation to other studies. The US system programming provided a sequence of methods to generate images including the standard elastogram, velocity inversion, and low-pass filtering. In addition, direct comparison of the results showed the proposed method effective in relation to these current techniques for generating shear waves and correcting images to obtain elastograms.

The routines defined in this study provide scope for future work. This includes add-ons such as reduced processing time for real time elastography imaging, incorporation of technologies applied to humans, aggregating algorithms for pulse sequences that eliminate the inconclusive regional, automatic selection of the best median array for image filtering, and/or use of specific transducers for clinical examinations (prostate, transvaginal, etc.).

Comparison of the images obtained from these methods demonstrated the fundamental objectives of this study: to assist in early diagnosis of tumors and to guide medical professionals and health institutions in treatment and accurate assessment of disease.
